# Genome-wide association analysis identify candidate genes for feed efficiency and growth traits in Wenchang chickens

**DOI:** 10.1186/s12864-024-10559-w

**Published:** 2024-06-28

**Authors:** Keqi Cai, Ranran Liu, Limin Wei, Xiuping Wang, Huanxian Cui, Na Luo, Jie Wen, Yuxiao Chang, Guiping Zhao

**Affiliations:** 1 Shenzhen Branch, Guangdong Laboratory for Lingnan Modern Agriculture, Genome Analysis Laboratory of the Ministry of Agriculture and Rural Affairs, Agricultural Genomics Institute at Shenzhen, Chinese Academy of Agricultural Sciences, Shenzhen, 518124 P.R. China; 2 State Key Laboratory of Animal Nutrition, Key Laboratory of Animal (Poultry) Genetics Breeding and Reproduction, Ministry of Agriculture, Institute of Animal Sciences, Chinese Academy of Agricultural Sciences, Beijing, 100193 P.R. China; 3 The Sanya Research Institute, Hainan Academy of Agricultural Sciences, Sanya, 572025 P.R. China; 4 Hainan (Tan Niu) Wenchang Chicken Co., LTD, Haikou, 570100 P.R. China

**Keywords:** Wenchang chicken, Feed efficiency trait, Growth trait, GWAS, Candidate gene

## Abstract

**Background:**

Wenchang chickens are one of the most popular local chicken breeds in the Chinese chicken industry. However, the low feed efficiency is the main shortcoming of this breed. Therefore, there is a need to find a more precise breeding method to improve the feed efficiency of Wenchang chickens. In this study, we explored important candidate genes and variants for feed efficiency and growth traits through genome-wide association study (GWAS) analysis.

**Results:**

Estimates of genomic heritability for growth and feed efficiency traits, including residual feed intake (RFI) of 0.05, average daily food intake (ADFI) of 0.21, average daily weight gain (ADG) of 0.24, body weight (BW) at 87, 95, 104, 113 days of age (BW87, BW95, BW104 and BW113) ranged from 0.30 to 0.44. Important candidate genes related to feed efficiency and growth traits were identified, such as *PLCE1, LAP3, MED28, QDPR, LDB2* and *SEL1L3* genes.

**Conclusion:**

The results identified important candidate genes for feed efficiency and growth traits in Wenchang chickens and provide a theoretical basis for the development of new molecular breeding technology.

**Supplementary Information:**

The online version contains supplementary material available at 10.1186/s12864-024-10559-w.

## Introduction

**Fig. 1:**
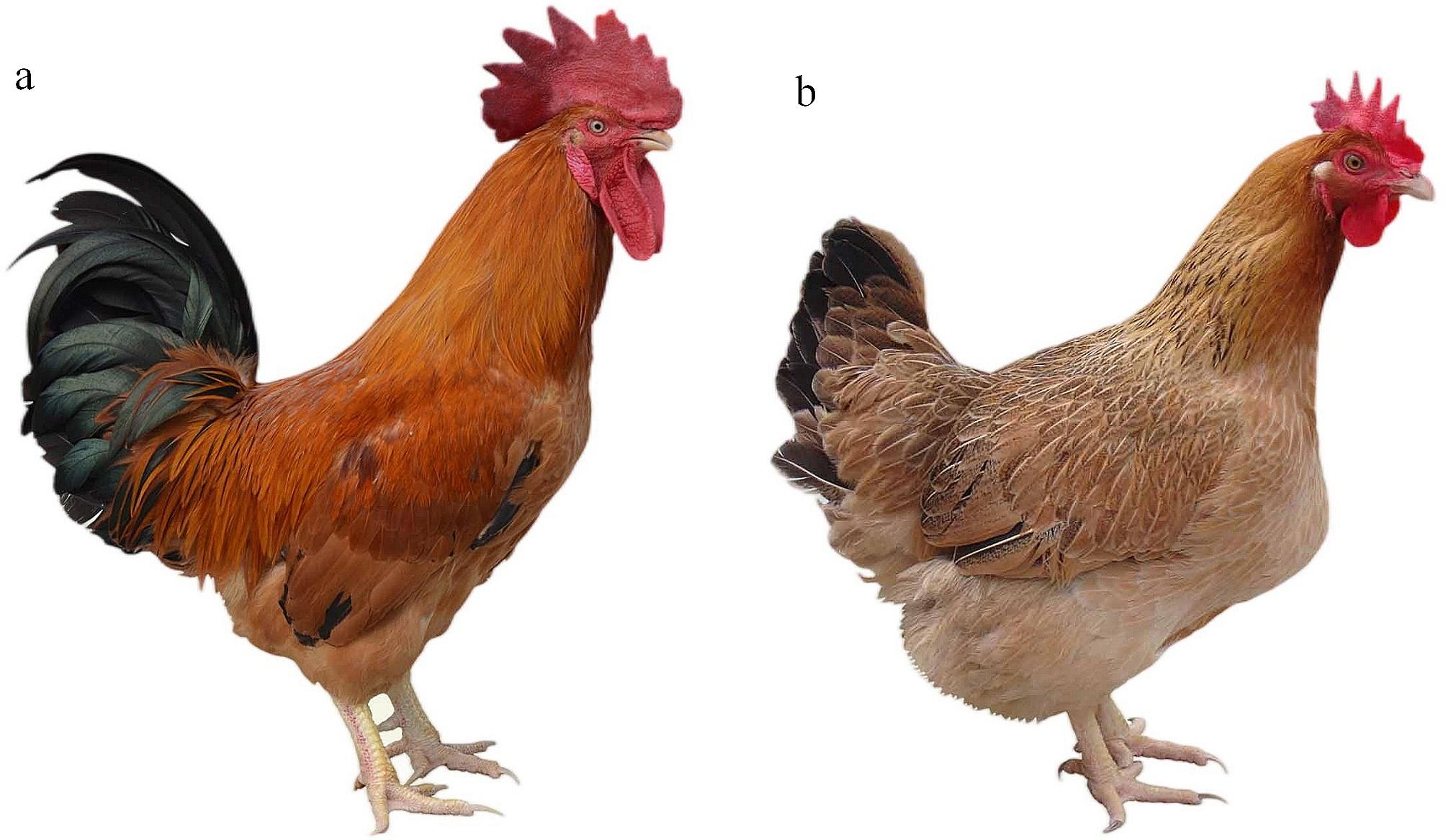
Picture for Wenchang chicken. (**a**), roosters (**b**), hens

Poultry production is an important entreprise worldwide. Chicken is considered a healthy white meat source that is lower in fat, calories and cholesterol than other red meat sources [1]. In recent years, the promotion of white meat consumption has gradually become a trend [2]. As an important part of the white meat market, chickens represent an efficient and inexpensive source of animal protein [3]. Since the 1980s, with the continuous development of modern broiler production, China has become the second largest country in the world in terms of chicken production and consumption [4]. The main breeds of chickens are native breeds and broilers [5]. Wenchang chicken (Fig. 1) is one of the most popular chicken breeds in the local chicken industry, originating from Hainan Island in the South China Sea and has been raised for 400 years. It is famous for its excellent meat quality and is one of the four famous dishes from Hainan [6]. It’s an economic mainstay of animal husbandry in Hainan Province, with an annual output of nearly 100 million chickens and a total output value of 1.78 billion dollars in 2020 [7].

However, the primary shortcoming of Wenchang chickens is their low feed efficiency [8]. Therefore, there is an urgent need to find a more reliable breeding method to improve the feed efficiency of Wenchang chickens. Feed represents more than 70% of the total cost of poultry production, and improving feed efficiency has consistently been the goal of any chicken breeding strategy [9]. Feed efficiency is contingent upon the relation between the feed intake (FI) and the growth (or bodyweight gain) of an animal and is quantified by several indexes, such as RFI and feed conversion rate (FCR) [10–12]. Feed efficiency is influenced by several factors, including the breed and its sex, age, diet, and management [13, 14].The RFI is an important index measuring feed efficiency, and it is defined as the difference between the actual and expected feed intake [15].In 1963, the concept of RFI was first proposed in beef cattle research [16], and it was first applied to chickens by Luiting in 1991 [17], covering the calculation methods of RFI, heritability calculation, and the phenotypic and genetic correlations with relevant traits. Since RFI reflects the variation in feed efficiency, it appears to be independent of growth traits [15]. Studies have shown that RFI is moderately heritable in poultry [12, 18]. The study by Bai et al. demonstrates that selecting for low RFI can improve poultry feed efficiency without compromising growth performance [19]. Growth traits are key in poultry breeding, and the properties of growth traits need to be considered in breeding for feed efficiency. In recent years, GWAS has been applied in poultry to explore the association between host genetics and economic traits [20]. Earlier studies identified candidate genes including *NSUN3*, *EPHA6*, and *AGK* for broilers RFI, while *LAP3* was identified as a candidate gene for broiler body weight [21–26]. By deeply studying the functions and regulatory mechanisms of these genes, our aim is to provide more efficient and sustainable solutions for the chicken breeding technologies [4].

In the breeding process of this study, the focus will be on maintaining the excellent meat quality while moderately improving the feed efficiency and growth rate. The objectives of this study were (1) to determine the inheritance pattern of feed efficiency and growth traits to provide a basis for formulating a better breeding method for Wenchang chickens, and (2) to find key variants affecting feed efficiency and growth traits of Wenchang chickens and also to elucidate the molecular mechanism of feed efficiency and growth traits.

## Materials and methods

### Experimental birds

The study uses 1,547 chickens hatched and raised by Hainan Tanniu Wenchang Chicken Co., Ltd. These birds are from a commercial line of Wenchang chicken selected for 18 generations. All birds were raised in three-tier battery cages (one bird per cage) under the same management and nutritional conditions. The diet was formulated based on the National Research Council (NRC) requirements [27] and the Feeding Standards of Chickens established by the Ministry of Agriculture, Beijing, China [28]. The chickens were raised from 87 to 113 days to collect data on individual phenotypes.

### Phenotypes

The phenotypes measured in this study included body weight and feed intake of chickens.

For body weight measurements, an electronic scale with a precision of 0.1 g was utilized. The trough was emptied of any remaining feed 12 h prior to weighing, and body weights were recorded at 87, 95, 104, and 113 days of age.

Feed intake in this experiment was recorded for 26 days. Fresh feed was added at a fixed time every day and recorded. In addition to the above measured phenotypes, the phenotypes calculated from the measured phenotypes included FI, ADFI, ADG, Metabolic weight in the middle of the test (MWT), and RFI.

FI, g.

1 $$\eqalign{& FI\hspace{0.17em}=\hspace{0.17em}feed\,amount\,during the\,test\hspace{0.17em}- \cr & \hspace{0.17em}Weight\,of\,feed\,remaining\,at\,the\,end\,of \,the\,test\cr}$$

ADFI, g/d.

2 $$\text{A}\text{D}\text{F}\text{I}=\text{F}\text{I} / 26$$

ADG, g/d.

3 $$\text{A}\text{D}\text{G}= (\text{B}\text{W}113-\text{B}\text{W}87) / 26$$

MWT, g.

4 $$\text{M}\text{W}\text{T}= \left[\right(\text{B}\text{W}87 + \text{B}\text{W}113) / {2]}^{0.75}$$

RFI, g/d: The RFI was obtained from the multiple regression equation of average daily feed intake and metabolic weight in the middle of the test and average daily weight gain [18]. The equation was as follows:

5 $$\text{R}\text{F}\text{I}=\text{A}\text{D}\text{F}\text{I}-({\upmu } + \text{s}\text{e}\text{x} + {\upbeta }1 \text{M}\text{W}\text{T} + {\upbeta }2 \text{A}\text{D}\text{G})$$

where ADFI represents the mean average daily feed intake, µ is the intercept, sex is a fixed effect, MWT and ADG are as defined above, β1 and β2 represent the partial regression coefficient.

### Genotyping, imputation, and quality control (QC)

At 112 days of age, 2 mL of blood was collected from the wing vein, placed in an anticoagulant tube containing EDTA-K2, mixed, and stored at -20℃ until later analysis. Genomic DNA was extracted from blood samples with the phenol-chloroform method. Genotyping was conducted with a customized chicken 55 K SNP array (Beijing Compass Biotechnology Co., Ltd., Beijing, China) [29]. QC of the generated genotype data was achieved using PLINK (V2.0) (https://www.cog-genomics.org/plink/2.0/) software. The specific process setting the individual genotype detection rate at ≥ 90%, single SNP detection rate at ≥ 90%, minimum allele frequency (MAF) of 95%, and retention of SNPs on autosomes 1–28. A total of 45,278 SNPs in 1,479 chickens (762 males and 717 females) passed the QC. Whole genome resequencing of 247 individuals from the 1,479 Wenchang chickens, including 25 males and 222 females was carried out. The sequencing generated 150 bp paired end reads on an Illumina NovaSeq 6000 platform with the average depth of approximately 10×, at the Shenzhen BGI Co., Ltd. The QC standards were as follows: setting the detection rate of individual genotype at ≥ 90%, the detection rate of single SNP site at ≥ 90%, a MAF of 95%, and Hardy-Weinberg equilibrium (HWE) at *P* < 0.000001. A total of 12,590,784 autosomal SNPs in 247 individuals were passed the QC.

Beagle 5.2 software was used to impute the 55 K chip data to the whole-genome sequence (WGS) level [30]. Before imputation, inconsistencies between the target panel and the reference panel were checked using conform-gt software (http://faculty.washington.edu/browning/conform-gt.html). Then, the 55 K SNP chip data were populated to the resequencing level using Beagle 5.2 software. The QC condition: HWE at *P* < 1.00e-6, setting the detection rate of individual genotype at ≥ 90%, the detection rate of single SNP site at ≥ 90%, and a MAF of ≥ 0.05. A total of 12,184,765 autosomal SNPs from 1,479 samples passed QC.

### Estimation of heritability and genetic correlations

Phenotypic correlation coefficients were calculated using ggpairs within the GGally R package, and then genetic parameter estimation was performed using restricted maximum likelihood (REML) of GCTA v1.93.2 beta software [31, 32]. The statistical model used was:

$$y = Xb+Z\alpha +e$$

6 $$\eqalign{y &= Xb + Za + e \cr Var\left[ {\matrix{\alpha \cr e \cr } } \right] &= \left[ {\matrix{{G\sigma _a^2} & 0 \cr 0 & {1\sigma _e^2} \cr } } \right] \cr}$$

where, $\text{y}$ is a vector of observations, $\text{b}$ is a vector of fixed effects (i.e., sex), ${\upalpha }$ is the random vector representing the genomic effects,$e$is the vector of random residual effects, $\text{X}$and $\text{Z}$ are incidence matrices. The distribution of the random animal effect $\alpha$ is $\alpha \ \sim N(0,\,{\rm{G}}\sigma _a^2)$ with $\text{G}$ is being the genomic relationship matrix, and ${\sigma }_{a}^{2}$ being the additive genetic variance.

### Genome-wide association study

A GWAS analysis was carried out between all the genotyped SNPs and feed efficiency and growth traits using a mixed linear model (MLM). The MLM for feed efficiency and growth traits was performed using sex (female or male) as a fixed effect and the top three principal components (PCs) as covariates. All association tests were performed using the MLM option in GCTA based on the following model [33]:

7 $$y = Xb + Z\mu + e$$

where y is a vector of observations, X and Z are incidence matrices for the vectors for parameters b and µ, b is a vector of fixed effects including the sex and three eigenvectors from principal component analysis (PCA), µ is the vector of the additive genetic effect of the candidate SNP to be tested for association, and e is the vector of the residual effect.

The Bonferroni correction method was used in this study to determine the significance thresholds, and the formula for performing the Bonferroni corrected multiple tests was as follows:

8 $$P=\alpha /N$$

Where $P$ is the corrected significance threshold,$\alpha$represents the significance threshold for a single test, and *N* represents the number of multiple hypothesis tests, i.e., the number of SNPs analysed by GWAS. We calculated the number of genome-wide independent markers using the PLINK (V1.9) command -indep-pairwise, with a window size of 25 SNPs, a step of five SNPs, and an r^2^ threshold of 0.2. Manhattan and quantile‒quantile (Q-Q) plots were derived from the GWAS results using the qqman (https://cran.r-project.org/web/packages/qqman/) and Cairo (http://www.rforge.net/Cairo/) packages within R software (http://www.r-project.org/). LD blocks of target regions were performed using Haploview v4.2 software [34]. For additive and dominance effects of important SNPs on traits, the calculation process in this study was done in ASReml v4.1 software (https://asreml.kb.vsni.co.uk/knowledge-base/asreml_documentation). The SNP positions were updated according to the newest release from Ensembl (https://asia.ensembl.org/index.html). Identification of the closest genes to genome-wide significant and suggestive variants was obtained using the ChIPpeakAnno package (https://www.bioconductor.org/packages/devel/bioc/vignettes/ChIPpeakAnno/inst/doc/pipeline.html). Gene function enrichment analysis was performed using bioinformatics (https://www.bioinformatics.com.cn).

### Differential expression analysis

In this study, the RFI or BW of 127 broiler chickens was ranked from low to high. Using individuals with the highest (*n* = 15) and lowest RFI (*n* = 15) phenotypes, and individuals with the highest (*n* = 15) and lowest (*n* = 15) BW. The Wenchang chickens used in this study were a mix of males and females, so we did not differentiate between genders in the 30 chickens selected. Gene expression of important candidate genes in the different groups can demonstrate the reliability of the GWAS results. Figures were generated using GraphPad Prism 8 [4].

## Results

### Description of phenotypic traits

**Fig. 2:**
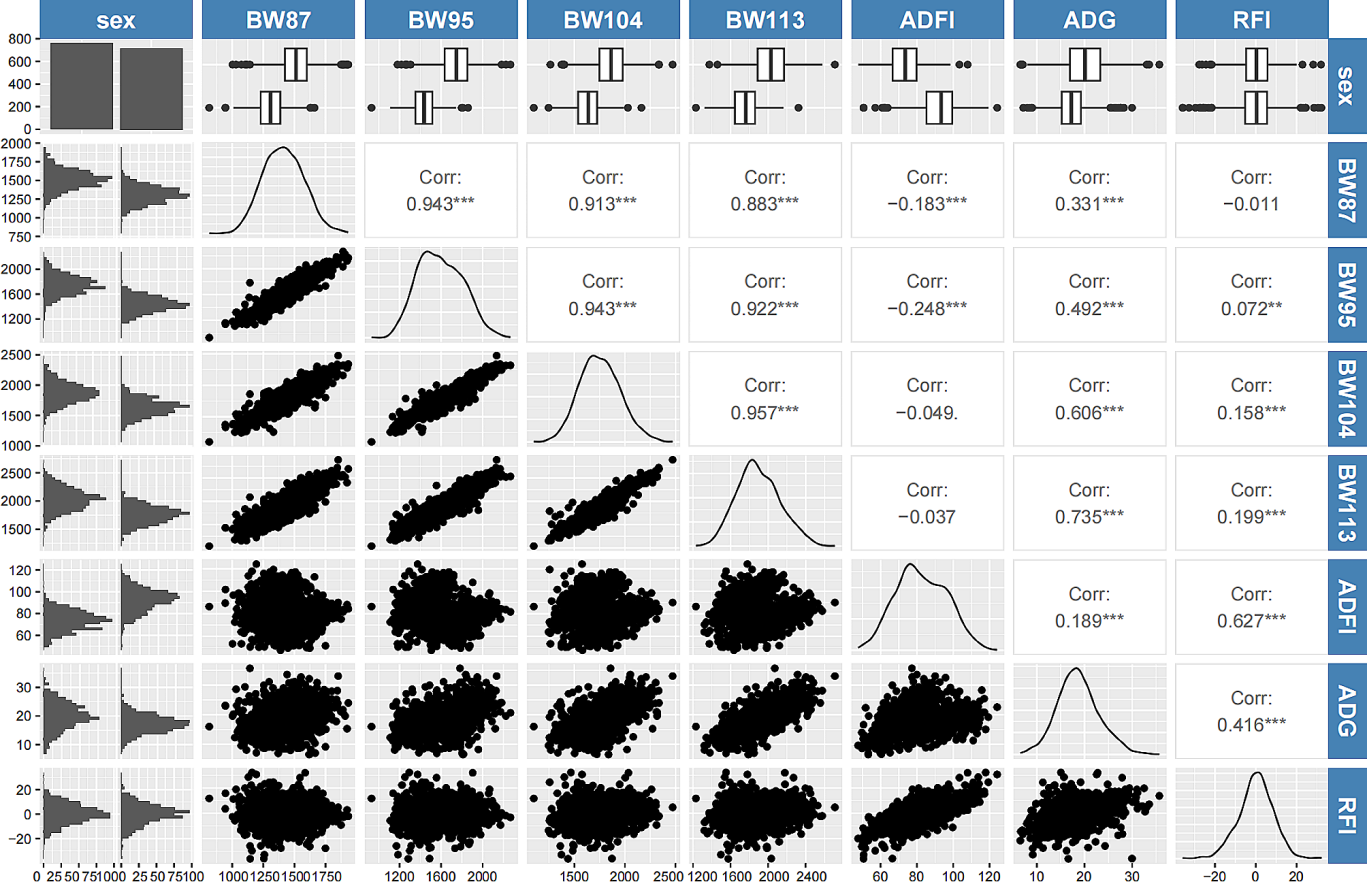
Phenotypic data and correlation analysis of Wenchang chicken

Descriptive statistics of the feed efficiency traits of Wenchang chickens are shown in Table 1; Fig. 2. According to the body weights at 87, 95, 104 and 113 days, Wenchang chickens exhibited slow growth, with an ADFI of 82.52 g/d and an ADG of 8.66 g/d. The RFI ranged from − 39.14 to 36.25 g/d.

**Table 1:** Descriptive statistics for feed efficiency and growth traits of Wenchang chickens (*N*^1^ = 1532)

Traits	M^2^	SD^3^	Min^4^	Max^5^	CV%^6^
BW87 (g)	1409.48	165.13	816.00	1933.00	11.72%
BW95 (g)	1590.81	214.57	930.00	2268.00	13.49%
BW104 (g)	1751.63	200.25	1099.00	2472.00	11.43%
BW113 (g)	1894.36	229.16	1231.00	2711.00	12.10%
ADFI (g/d)	82.52	13.85	47.58	124.19	16.78%
ADG (g/d)	18.66	4.35	6.54	35.69	23.31%
RFI (g/d)	-1.78E-13	13.17	-39.14	36.25	——

^1^Number of animals (*N*), ^2^mean (M), ^3^standard deviation (SD), ^4^maximum (Max), ^5^minimum (Min), and ^6^coefficient of variation (CV) of Wenchang chickens

### Genetic parameters’ estimates

**Fig. 3:**
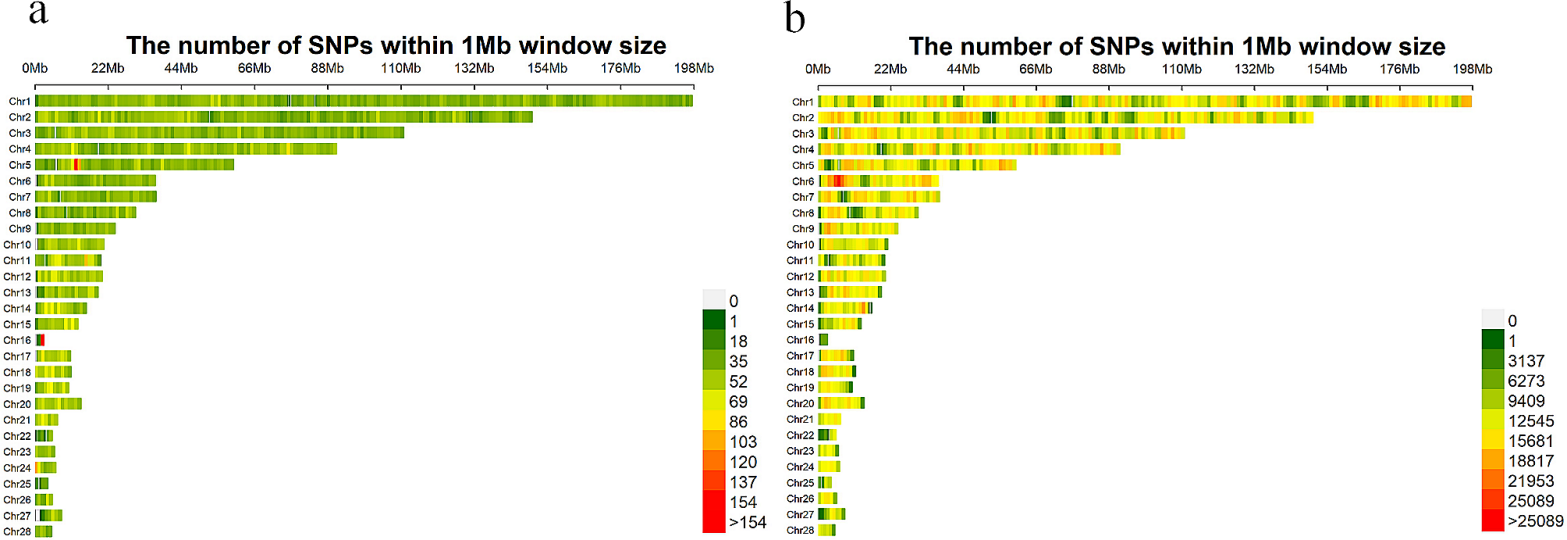
Comparison of density markers before and after the imputation

Comparison of density markers before and after the imputation in Fig. 3 revealed a significant increase in loci after imputation. The estimated genetic parameters of residual feed intake and body weight are shown in Table 2. The heritability of the RFI, BW, ADFI and ADG ranged from moderate and low level (0.05–0.44). RFI had the highest genetic association with ADFI and ADG, at 0.92 and 0.86, respectively (*P* < 0.001). Regarding phenotypic correlation, the genetic correlation between RFI and ADFI was 0.63 and decreased with body weight to a minimum of 0.20. Based on the result, the heritability of RFI in Wenchang chicken is low, while the heritability of body weight is not significantly different from that of other breeds.

**Table 2:** Estimates of genetic parameters for feed efficiency and growth traits of Wenchang chickens

	RFI	ADG	ADFI	BW87	BW95	BW104	BW113
RFI	**0.05±0.03**						
ADG	0.86±0.27	**0.24±0.05**					
ADFI	0.92±0.11	0.95±0.05	**0.21±0.04**				
BW87	0.65±0.26	0.77±0.09	0.86±0.07	**0.30±0.05**			
BW95	0.67±0.25	0.79±0.07	0.89±0.06	0.99±0.01	**0.40±0.05**		
BW104	0.77±0.26	0.89±0.05	0.95±0.04	0.97±0.01	0.98±0.01	**0.39±0.05**	
BW113	0.80±0.25	0.92±0.03	0.97±0.03	0.96±0.02	0.96±0.01	1.00±0.00	**0.44±0.05**

The genetic correlation was below the diagonal and heritability of feed efficiency and growth traits were on the diagonal. The superscript ** means *P* < 0.01, *** means *P* < 0.001

### Genome-wide association study of feed efficiency and growth traits

**Fig. 4:**
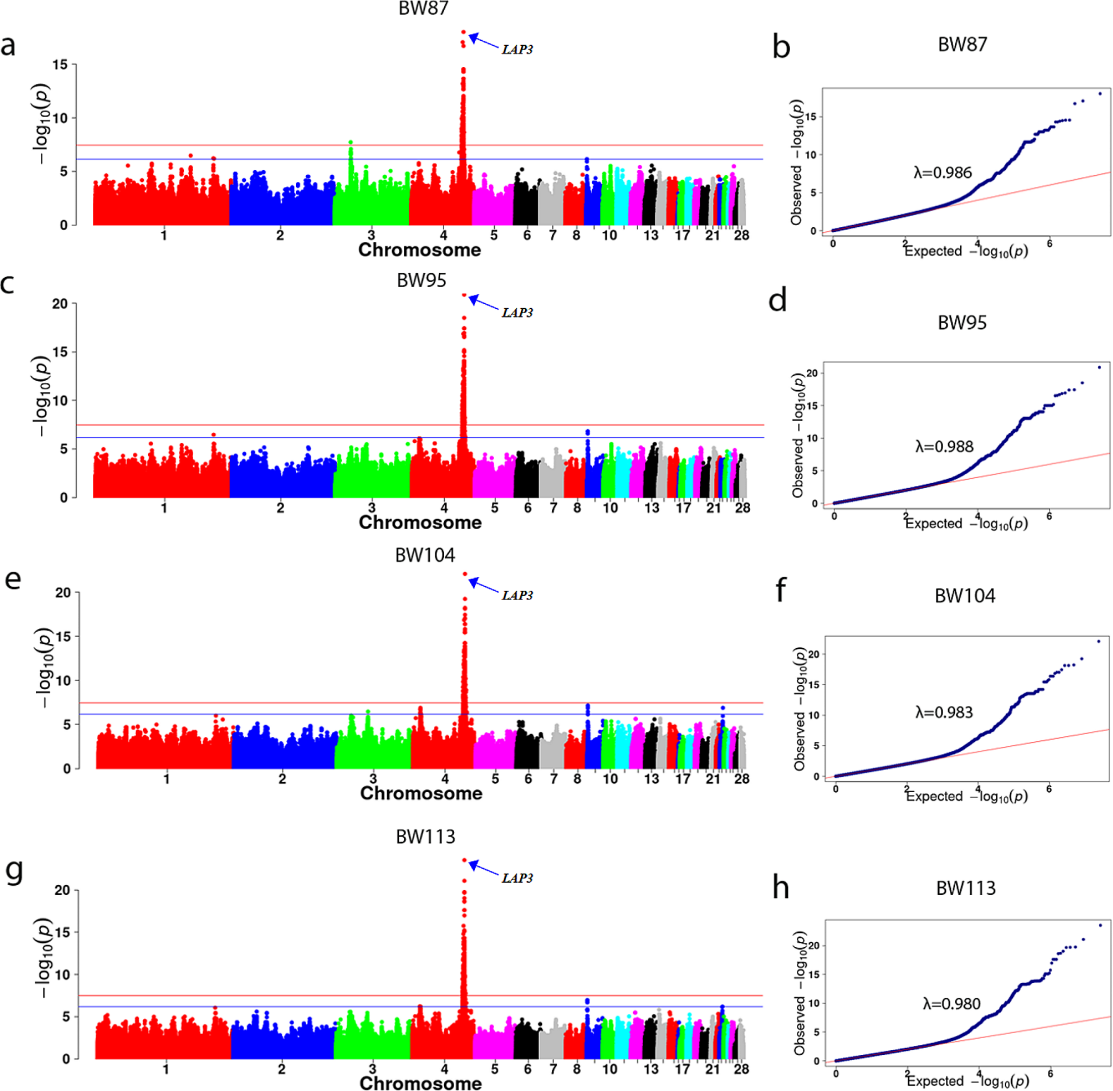
Manhattan and Q-Q plots of GWAS for feed efficiency traits. The horizontal red lines indicate the thresholds for genome-wide significance (*P* = 3.47E-08), and the horizontal blue lines indicate the thresholds for suggestive significance (*P* = 6.93E-07)

The GWAS results showed significant SNPs on GGA 2, 6 and 26 were associated with RFI (expansion coefficient λ of 0.987) (Fig. 4a-b). One of significant SNPs 6_21123592 on GGA6 was located on the intron of the *PLCE1* gene, with this SNP explaining 2.46% of the genetic variation. Additionally, significant SNPs 2_45795056 and 26_2851843 were located in the lncRNA introns of the *ENSGALG00000052614* and *ENSGALG00000001264* genes, respectively (Table 3). The GWAS results showed significant SNPs on GGA 2, 4, 6, 19, and 28 were associated with ADFI (expansion coefficient λ of 0.994) (Fig. 4c-d). Specifically, the significant SNPs 4_75971941, 4_85488222, 19_2763444, and 28_388715 were found on the genes *LAP3, IMMT, GATSL2*, and *FBN3*, respectively (Table 3). The SNP 6_21123592 for RFI in the CT genotype exhibited significantly higher values than those in the CC genotype (Table 4). Another SNP 2_45795056 showed a significant additive effect on ADFI but a nonsignificant effect on the two feed efficiency traits assessed in this study. The estimated additive effect of candidate SNP 6_21123592 on RFI and ADFI were − 0.81 and 2.67 respectively, while the dominance effect was − 2.99 and 0.93 respectively. Significant dominance effects were observed on both RFI and ADFI, while the additive effect was not significant (Table 5).

**Table 3:** The significant SNPs information of the feed efficiency traits in Wenchang chicken

Trait	GGA	Position	Ref /Alt	Variant ID	*P* value	Gene	Annotation
**RFI**	2	45,795,056	T/G	rs15094873	4.22E-07	*ENSGALG00000052614*	intron variant
	6	21,123,592	C/T	rs3386308535	6.39E-07	*PLCE1*	intron variant
	26	2,851,843	G/A	rs313737158	6.64E-07	*ENSGALG00000001264*	intron variant
**ADFI**	2	45,795,056	T/G	rs15094873	2.51E-07	*ENSGALG00000052614*	intron variant
	4	75,971,755	T/C	rs80678404	9.97E-08	*LAP3*	intron variant
		75,971,941	T/C	rs80610898	3.83E-08		intron variant
		75,974,422	C/T	rs731292712	1.18E-07		missense variant
		85,488,222	A/G	rs314508466	6.78E-07	*IMMT*	intron variant
	6	19,485,905	A/G	rs3386252369	2.20E-07	*ENSGALG00000027353*	intron variant
	19	2,763,444	G/A	rs1058587386	2.19E-07	*GATSL2*	intron variant
	28	388,715	A/G	rs312632010	5.57E-07	*FBN3*	intron variant
**ADG**	4	75,971,279	T/C	rs735081553	4.40E-07	*LAP3*	3 prime UTR variant
		75,971,373	C/T	rs80690160	7.43E-09		3 prime UTR variant
		75,971,584	G/A	rs80771106	4.40E-07		synonymous variant
		75,971,617	C/T	rs10729884	6.28E-09		synonymous variant
		75,971,755	T/C	rs80678404	1.21E-08		intron variant
		75,971,941	T/C	rs80610898	4.66E-10		intron variant
		75,974,422	C/T	rs731292712	3.38E-09		missense variant

**Table 4:** Effect of candidate SNPs on feed efficiency traits

SNP	Genotypes	RFI (g/d)	ADFI (g/d)
2_45795056	CC(*n*=812)	0.79±7.34^a^	83.43±13.90^a^
	CT(*n*=554)	-0.62±8.28^b^	81.68±13.76^ab^
	TT(*n*=113)	-2.00±7.10^b^	79.68±13.53^b^
6_21123592	CC(*n*=1303)	-0.36±7.71^a^	82.02±13.81^a^
	CT(*n*=174)	3.11±7.34^b^	85.94±13.96^b^
	TT(*n*=2)	1.25±4.14^ab^	88.00±1.85^ab^

The different superscripts mean the significant difference, namely *P* < 0.05

**Table 5:** Additive and dominance effects of candidate SNPs on feed efficiency traits^1^

SNP	Effect size ^2^	RFI (g/d)	ADFI (g/d)
2_45795056	additive	1.39±0.12	-0.01±1.06**
	dominance	1.88±0.19	0.13±0.04
6_21123592	additive	-0.81±1.79	2.67±1.42
	dominance	-2.99±5.98**	0.93±6.13**

^1^ The superscript * means *P* < 0.05, ** means *P* < 0.01

^2^ The additive effect (a) was calculated as: (AA-BB)/2, the dominance effect (d) was calculated as: AB-[(AA+BB)/2], AA and BB represent the respective homozygous genotypes, while AB represents the heterozygous genotype

**Fig. 5:**
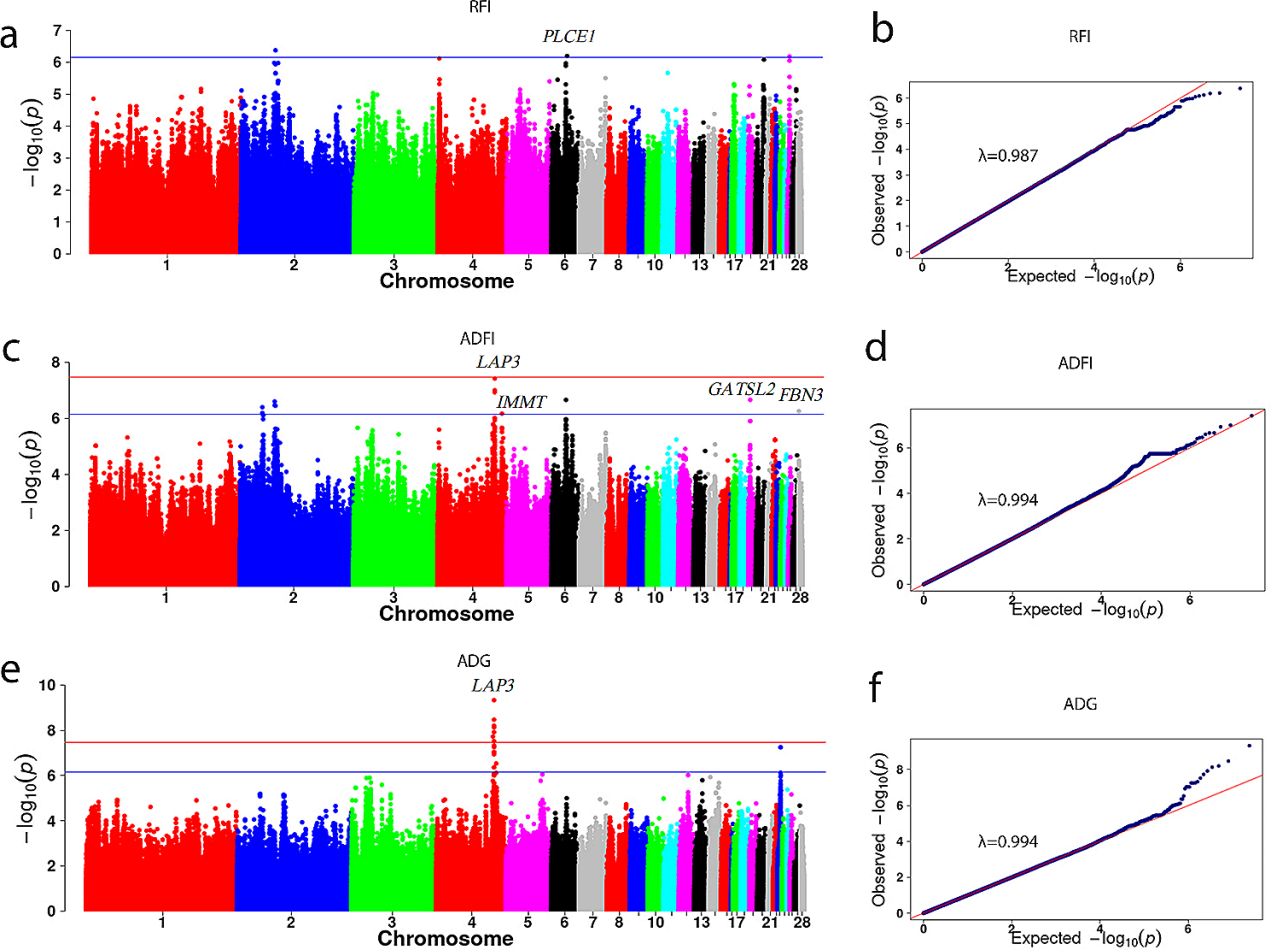
Manhattan and Q-Q plots of GWAS for growth traits. The horizontal red lines indicate the thresholds for genome-wide significance (*P* = 3.47E-08), and the horizontal blue lines indicate the thresholds for suggestive significance (*P* = 6.93E-07)

The GWAS analysis results for growth traits at different ages are presented in Fig. 5; Table 6. Significant QTLs affecting BW, ADG, and ADFI were observed in the 73.15–76.55 Mb interval of GGA4. Specifically, BW87 (Fig. 5a, Table S1) had 661 SNPs in the significant interval, BW95 had 933 SNPs (Fig. 5c, Table S2), BW104 had 1018 SNPs (Fig. 5e, Table S3), BW113 had 1150 SNPs (Fig. 5g, Table S4), and ADFI and ADG had 3 and 7 SNPs (Table 3; Fig. 4c and e). The most prominent SNP among these traits was 4_75971941, located in the intron of the LAP3 gene. There were 13 important candidate genes in the colocalization interval of different day-ages, including *LAP3, KCNIP4, NCAPG, LDB2, FAM184B, SEL1L3, ZCCHC4, PPARGC1A, PACRGL, SLIT2, LCORL, MED28* and *QDPR* (Table 6). Through gene function enrichment analysis (Figure S1), we identified significant enrichment primarily in functions such as RNA biosynthetic process, regulation of RNA metabolic process, and regulation of cellular macromolecule biosynthetic process.

**Table 6:** The significant SNP information of growth traits in Wenchang chicken

Trait	GGA	Base-pair region		*n* SNP	Lead SNP	Base pair	Alleles	*P*-value	Candidate
		Start	End						
BW87	4	73,161,256	76,324,041	661	rs80610898	75,971,941	T/C	9.80E-19	*LAP3,FAM184B, KCNIP4,LCORL, LDB2,MED28,NCAPG, PACRGL, PPARGC1A, QDPR, SEL1L3,SLIT2,ZCCHC4*
BW95	4	73,165,463	76,330,595	933	rs80610898	75,971,941	T/C	1.28E-21	
BW104	4	73,155,109	76,330,595	1018	rs80610898	75,971,941	T/C	8.11E-23	
BW113	4	74,768,255	76,559,941	1150	rs80610898	75,971,941	T/C	3.02E-24	

**Fig. 6:**
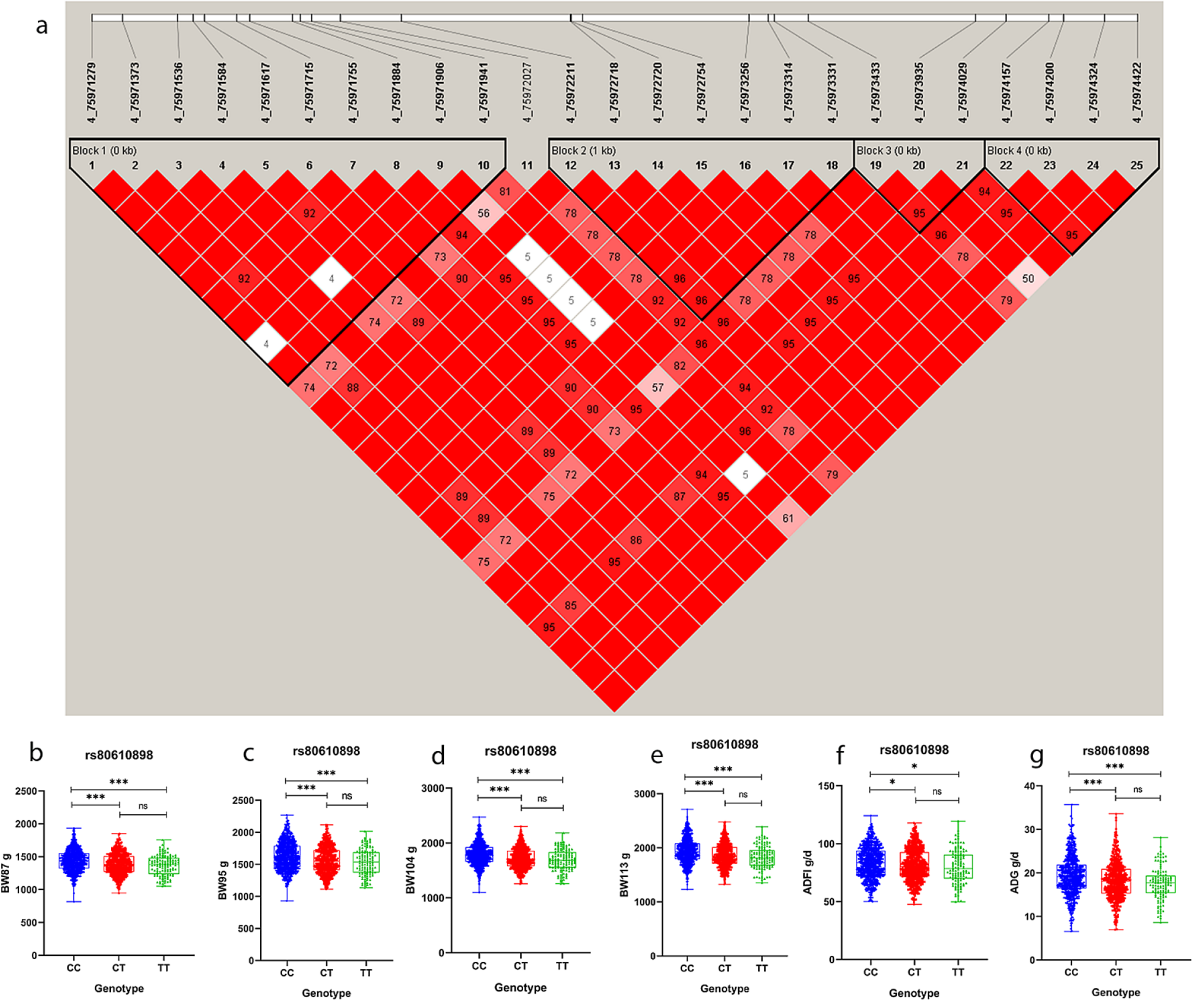
Association results of the candidate SNPs on GGA4. (**a**), LD analysis of the 25 significant SNPs on GGA4. (**b**, **c**, **d**, **e**, **f**, **g**), The phenotypic differences of individuals with different genotypes at rs80610898 on GGA4. *, *P* < 0.05; **, *P* < 0.01; ***, *P* < 0.001; ns, no significance

The four LD blocks were detected within the common location of growth traits on GGA4:75971.28-75974.42 kb (Fig. 6), each containing 3–10 SNPs. The most significant SNP 4_75971941 was located on block1, which is located in the intron of the *LAP3* gene. Genotype analysis showed that the body weight of the CC genotype at different ages was significantly higher than that of the CT genotype. Specifically, the weight of the CC genotype for BW87 was 54.89 g heavier than that of the CT genotype, and the weight of the CC genotype for BW113 was 82.80 g heavier than that of the CT genotype (Fig. 6b-g). The estimated additive effects of candidate SNP 4_75971941 on body weight at different ages were 37.97, 46.67, 53.38 and 60.53, while the dominance effects were − 16.92, -16.19, -17.98 and − 22.26. SNP 4_75971941 had significant additive effects on BW and ADG ranging from 37.97 to 60.53. The additive effects increased with age, while the locus had significant dominance effects on BW and nonsignificant dominance effects on ADG and ADFI (Table 7).

**Table 7:** Additive and dominance effects of candidate SNPs on ADFI and growth traits

SNP	Effect size	BW87(g)	BW95(g)	BW104(g)	BW113(g)	ADFI (g/d)	ADG (g/d)
4_75971941	additive	37.97±8.31**	46.67±8.75**	53.38±5.19**	60.53±13.15**	1.91±-0.29	0.87±0.38**
	dominance	-16.92±1.68*	-16.19±-2.1*	-17.98±-3.69*	-22.26±0.00*	-0.26±-0.16	-0.21±0.16

The superscript * means *P* < 0.05, ** means *P* < 0.01

The candidate sites for body weight were determined to be located in distinct LD blocks. In this study, haplotype association analysis was conducted using the glm model with sex as a fixed effect (Table 8). The analysis indicated that block1 had a highly significant effect (*P* < 0.01) on BW at various ages, while block2 exhibited a significant effect (*P* < 0.05) on BW across different age groups. The results revealed that block2, block3, and block4 all had a significant impact on ADFI (*P* < 0.05), while none of the four LD blocks showed a significant impact on RFI (*P* > 0.05).

**Table 8:** The effect of different LD blocks on feed efficiency and growth traits in Wenchang chickens

Block (*P*)	*n* SNPs	BW87	BW95	BW104	BW113	ADFI	ADG	RFI
block1	10	1.51E-05	1.57E-11	1.39E-07	3.17E-06	5.73E-02	1.12E-01	3.96E-01
block2	7	5.60E-04	5.15E-05	3.20E-02	2.86E-02	3.25E-04	1.41E-01	6.26E-01
block3	3	2.83E-01	2.04E-01	4.37E-01	5.03E-01	1.13E-03	2.85E-02	3.74E-01
block4	4	4.93E-02	8.00E-03	1.78E-01	2.00E-01	3.45E-03	1.27E-01	8.76E-01

A significant impact on traits is indicated by *P* < 0.05 for LD blocks

### Differential expression analysis

**Fig. 7:**
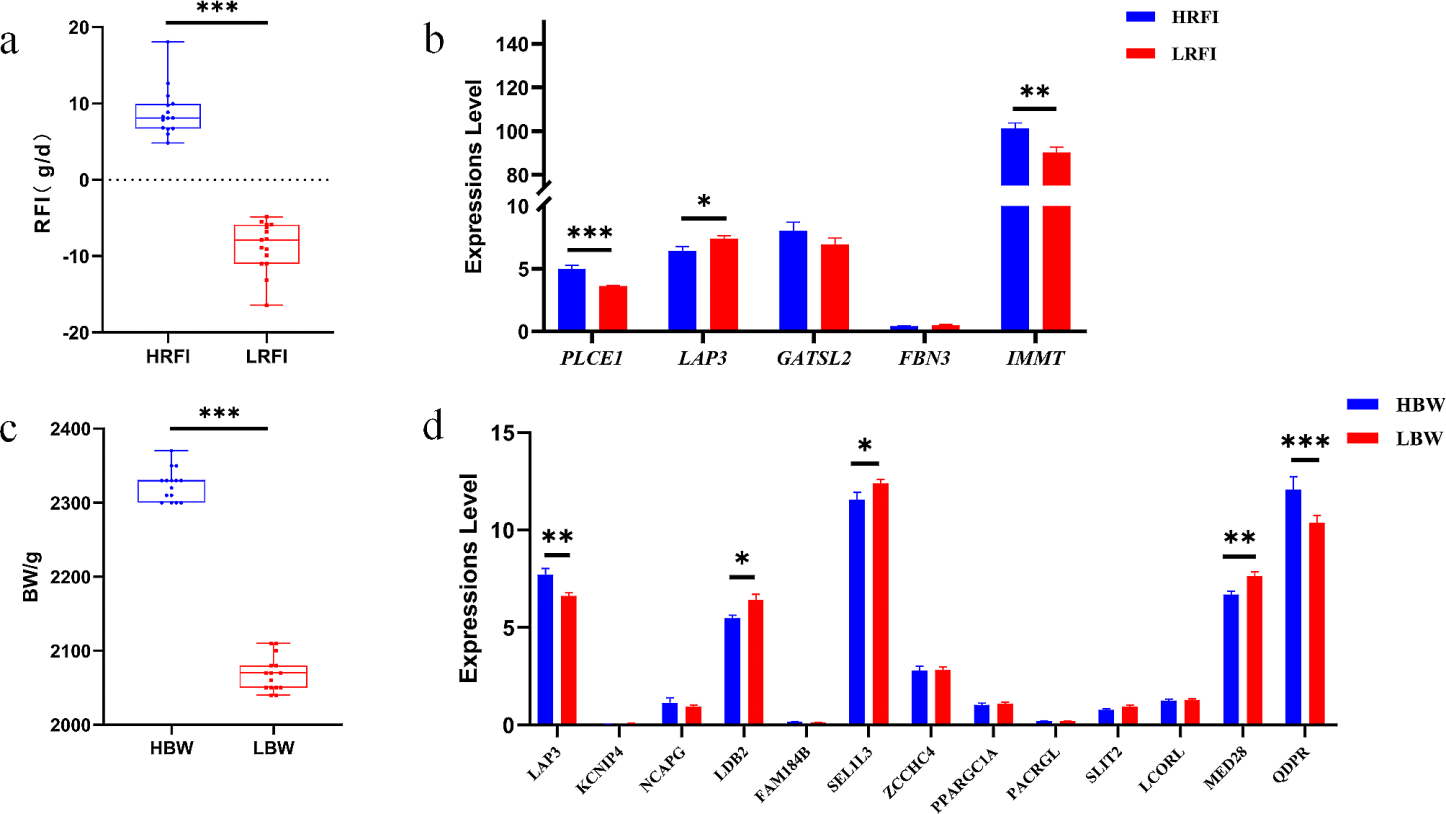
The expressions of candidate genes for feed efficiency and growth traits. (**a**), Analysis of RFI Differences between high and low RFI groups. (**b**), Differential expression of related candidate genes between high and low RFI groups. (**c**), Analysis of BW Differences between high and low BW groups. (**d**), Differential expression of related candidate genes between high and low weight groups. Data were expressed as the mean ± SEM (*n* = 15), *** *P <* 0.001, ** *P <* 0.01, * *P* < 0.05

We conducted gene differential expression analysis on all genes mapped to the feed efficiency and growth traits, respectively. Significant differences in phenotypes were observed between the high and low RFI groups (Fig. 7a) and between the high and low BW groups (Fig. 7c). Between the high and low RFI groups, the *PLCE1* and *IMMT* genes showed significantly (*P* < 0.01) higher expression levels in the high RFI group than in the low RFI group (Fig. 7b). Similarly, expression level of *LAP3, MED28*, and *QDPR* genes was significantly higher in the high BW group than that in the low BW group (*P* < 0.01), while expression of *LDB2* and *SEL1L3* genes was significantly (*P* < 0.05) higher in the low BW group than the high BW group (Fig. 7d).

## Discussion

### Phenotypic and estimation of the genetic parameters of Wenchang chickens

Body weight traits (BW87-BW113) of Wenchang chickens were selected for the GWAS analysis because Wenchang chickens are listing age within that age range. Through different trait measurements at this stage, our study revealed that the BW87 was 1,409.48 g, that of BW113 was 1,894.36 g, the ADG was 18.66 g/d, and the relative growth rate was 25.60%. These findings are consistent with previous studies on yellow-feathered chickens in China [35, 36]. The low heritability of RFI may be caused by the lack of systematic breeding efforts and the chickens are in the latter stage of growth, similar to the results of previous studies [37, 38]. Heritability of BW, ADFI and ADG revealed in the current study ranged from medium to low (0.21–0.44), which was similar to that of other breeds [39–41]. The phenotypic and genetic correlations between RFI and ADFI were 0.92 and 0.63, similar to the research of Shirali et al [42]. To optimize breeding outcomes, it is recommended to integrate additional metrics, such as ADFI, and implement a multi-trait selection approach throughout the breeding process, ultimately enhancing breeding results in Wenchang chickens.

### Genome-wide association study of feed efficiency and growth traits

To investigate the genetic architecture of feed efficiency and growth traits in Wenchang chickens, a mixed linear model was used for GWAS analysis of related traits. Through GWAS analysis, we identified significant SNP 6_21123592 on GGA6 was located within the intron of the *PLCE1* gene associated with RFI. Previous studies have shown that *PLCE1* gene is highly expressed in the nervous system and belongs to the phosphoinositide-specific phospholipase C family. The production of second messenger molecules such as diacylglycerol is regulated by activated phosphatidylinositol-specific phospholipase C enzymes, which mediate small GTPases of the Ras superfamily through the activity of its Ras guanine exchange factor. As the effector of heterotrimer and small G protein, *PLCE1* is involved in regulating cell growth, T-cell activation, actin organization and cell survival. Mapping the *PLCE1* gene function were mostly related to nervous system activity, which regulates the function of the brain in different ways, and the brain was key to regulating diet behavior and body energy homeostasis [43–46].

The significant QTL affecting BW, ADG, and ADFI was located in the 73.15–76.55 Mb interval of GGA4.There were 13 important candidate genes in the colocalization interval related to BW, including *LAP3, KCNIP4, NCAPG, LDB2, FAM184B, SEL1L3, ZCCHC4, PPARGC1A, PACRGL, SLIT2, LCORL, MED28* and *QDPR*. Among these candidates *LAP3, MED28, QDPR, LDB2* and *SEL1L3* demonstrated differential expression between high and low groups. Comparison with the Animal QTL database (Chicken QTL Database at (animalgenome.org)) reveals a total of 305 QTLs related to BW and 221 QTLs associated with ADG within 73.15–76.55 Mb interval of GGA4. *LAP3* has been shown to catalyze the hydrolysis of the amino-terminal leucine residues of protein or peptide substrates, with diverse functions in mammals, invertebrates, microbes, and plants [47]. The primary function of *LAP3* lies in protein maturation and degradation, processes crucial for metabolism, development, adaptation, and repair [48]. *LAP3* gene variation may underlie variations in growth rates among species and significant genetic polymorphism of traits of interest in breeding, potentially leading to applications in animal breeding. Furthermore, studies have been conducted on its SNP and its association with growth traits in mammals, such as bovine [49]. Another study found that the *LAP3* gene may have a potential function affecting muscle development in sheep [50]. Prenatal development stages are directly related to the growth and development of individual skeletal muscle, which determines the number of muscle fibers and postnatal muscle mass and further exerts long-term effects on the postnatal growth of animals [51–53]. Related research that tracked *LAP3* mutations in Hu sheep populations reportedly linked, the mutations with body weight at different growth stages [54]. In poultry *LAP3* gene was found to be associated with chicken growth traits [22].

Related study linked the *MED28* gene with live weight in sheep [55].We found that the *MED28* gene was related to muscle development in pig [56]. *QDPR* for an enzyme that regulates tetrahydrobiopterin (BH4), a cofactor for enzymes involved in neurotransmitter synthesis and blood pressure regulation. Therefore, *QDPR* gene are also important genes in the regulation of growth [57]. Previous studies have shown that the *LDB2* gene located at GGA4 is important for chicken growth traits, and a 31-bp indel was significantly correlated with multiple growth and carcass traits in the F2 population and affected the expression of the *LDB2* gene in muscle tissue [26, 58, 59]. It was also identified as an important candidate gene for rapid growth in chickens and had the strongest association with late body weight in Jinghai yellow chicken hens [22, 60, 61]. According to relevant studies, the *KCNIP4* gene is located on GGA4, and the candidate gene belongs to the potassium channel interaction protein family and has a wide range of physiological functions, including heart rate regulation, insulin secretion, neurotransmitter release, and smooth muscle contraction. It was considered to be an important candidate gene for growth traits of chickens. In addition, it was reported in different breeds and different growth stages, which also verified that the *KCNIP4* and *FAM184B* genes can affect the growth and development of chickens [58, 62, 63]. Studies have shown that the *NCAPG* gene is an important candidate gene for mammalian body size growth traits in growth trait association analysis of horses, sheep, and domestic donkeys and is involved in chromosome condensation and methylation [64–67]. The *FAM184B* gene has been found in previous studies to be associated with cattle carcass weight [68].

### Differential expression analysis

Transcriptomic data enable the quantification of DNA or RNA abundance and expression levels [69]. Differential analysis between different groups is conducted by measuring the expression levels of gene RNA, thereby identifying distinct patterns and variations in gene expression among different groups. The results of related studies also confirm gene expression data of important candidate genes in different groups can demonstrate the reliability of the GWAS results [70, 71]. We showed that expression of *PLCE1* in the high and low RFI groups of broilers, validatied it a key candidate gene for RFI. We found that the candidate gene *LAP3* was related to the BW, ADFI and ADG traits, as demonstrated by the significant difference in the expression of *LAP3* in high and low body recombination in broilers, and relevant research reports also indicated that this might be an important candidate gene affecting growth traits. This study also found that the candidate genes *LDB2*, *KCNIP4*, *FAM184B*, and *NCAPG* were located on GGA4 and were related to growth traits, which provides an important reference value for subsequent research on the growth traits of Wenchang chickens.

The validity of the SNPs and candidate genes obtained in this study is worth further extensive verification. The significant SNPs and candidate genes identified in this study can be incorporated into the chip markers in future research. By utilizing genomic selection breeding techniques, these markers can be used to breed and improve the target traits of WenChang chickens, ultimately enhancing breeding efficiency.

## Conclusions

In this study, we identified that the significant SNP 6_21123592 was located in candidate gene *PLCE1* for feed efficiency traits of Wenchang chickens, and the significant SNP 4_75971941 was located in candidate gene *LAP3.* Other candidate genes including *MED28, QDPR, LDB2*, and *SEL1L3* were identified for growth traits of Wenchang chickens. This provides a good theoretical basis for developing methods of Wenchang chickens breeding, and by further studying the functions and regulatory mechanisms of these genes, we could provide more efficient solutions for breeding of these chickens.

### Electronic supplementary material

Below is the link to the electronic supplementary material.

Supplementary Material 1

Supplementary Material 2

Supplementary Material 3

Supplementary Material 4

Supplementary Material 5

Supplementary Material 6

## Data Availability

The raw sequence data reported in this paper have been deposited in the Genome Sequence Archive (Genomics, Proteomics & Bioinformatics 2021) in National Genomics Data Center (Nucleic Acids Res 2022), China National Center for Bioinformation / Beijing Institute of Genomics, Chinese Academy of Sciences that are publicly accessible at https://bigd.big.ac.cn/gsa/browse/CRA016976.
