# Supplementary figures and images for: Genome-wide association analysis identify candidate genes for feed efficiency and growth traits in Wenchang chickens
Source: BMC Genomics. 2024 Jun 28;25:645. doi: 10.1186/s12864-024-10559-w (PMC11212279; doi:10.1186/s12864-024-10559-w)

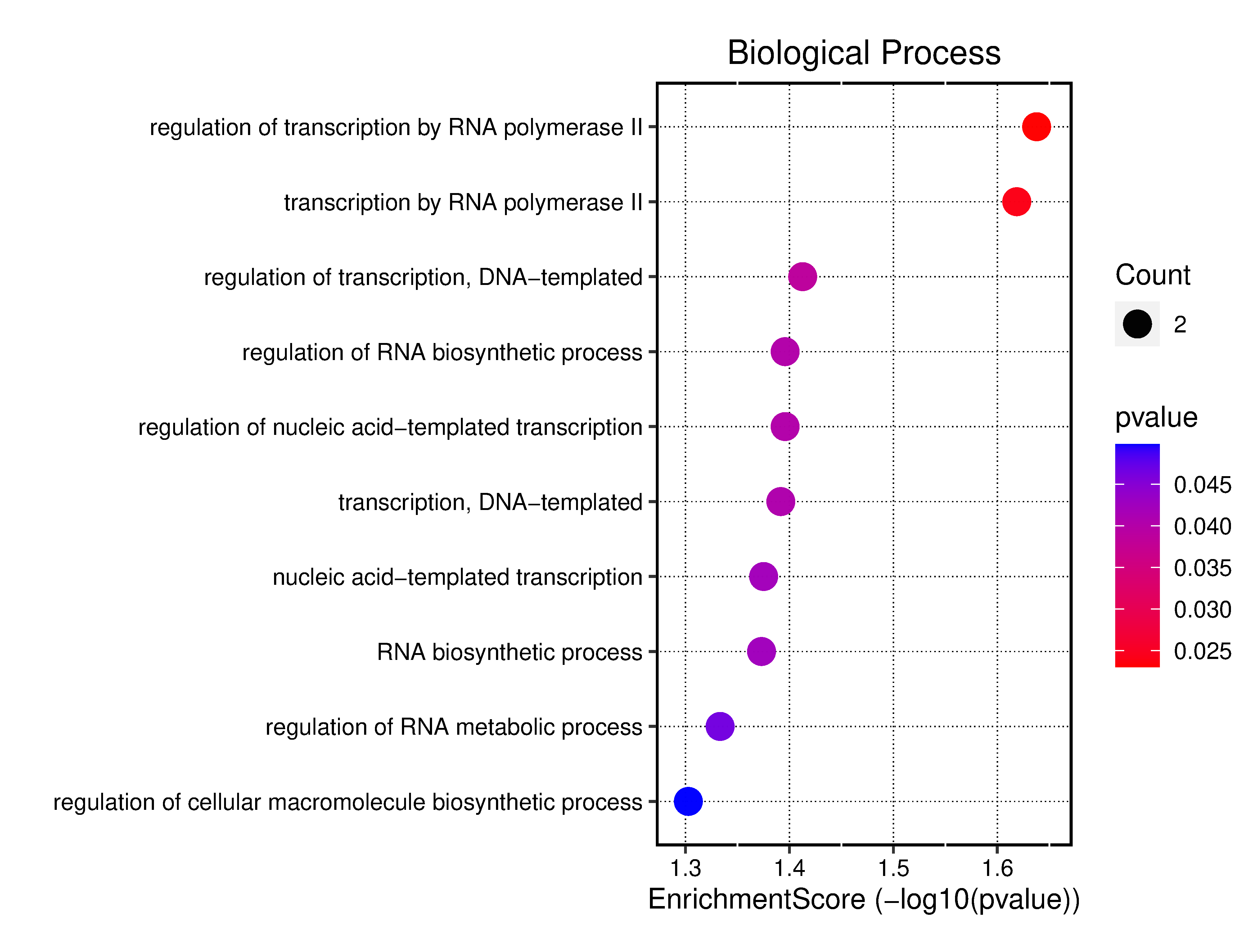

Supplement: Supplementary file 6 — Supplementary Material 6 [file 12864_2024_10559_MOESM6_ESM.tif]
